# Triterpenoids from *Ganoderma lucidum* inhibit cytochrome P450 enzymes interfering with the metabolic process of specific clinical drugs

**DOI:** 10.3389/fphar.2024.1485209

**Published:** 2024-11-15

**Authors:** Dawei Li, Yuxin Lin, Xia Lv, Yuzhuo Wu, Chaoyan Han, Peng Cao, Guixin Zhang, Aijing Leng, Jian Zhou, Chao Wang

**Affiliations:** ^1^ The First Affiliated Hospital of Dalian Medical University, Dalian, China; ^2^ College of Integrative Medicine, College of Pharmacy, Dalian Medical University, Dalian, China; ^3^ Department of Pharmacy, Dandong Frist Hospital, Dandong, China; ^4^ Department of Neurosurgery, General Hospital of Northern Theater Command (General Hospital of Shenyang Military Command), Shenyang, China; ^5^ Department of Pharmacy, The First Affiliated Hospital, Jiangxi Medical College, Nanchang University, Nanchang, China

**Keywords:** Ganoderma lucidum, functional food, CYP450s, lanostrane triterpenoid, drug-drug interactions

## Abstract

**Background:**

*Ganoderma lucidum* (*G. lucidum*), which possesses various biological effects, has been widely used as traditional medicine and functional food in Asian countries, especially China. In consideration of its various biological effects on human healthcare, *G*. *lucidum* was usually used in combination with other drugs. However, the potential drug-drug interaction induced by *G*. *lucidum* through cytochrome P450 enzymes (CYPs) remain unknown.

**Methods:**

Using the *in vivo* activity assay of CYPs, the inhibitory effects of *G. lucidum* and its constituents could be evaluated. The interference of *G. lucidum* on the metabolic processes of clinical drugs could be investigated. The chemical constituents of *G*. *lucidum* could be identified using LC-MS. The interaction between bioactive compounds and CYPs could be proposed through *in silico* docking analysis and molecular dynamics.

**Results:**

The dichloromethane extract of *G*. *lucidum* could inhibit various CYP450 subtypes *in vitro* and interfere with the pharmacokinetics of four drugs in rats. Triterpenoids were identified as the main constituents of the dichloromethane extract by Q-TOF-MS^n^ in preliminary analyses. Then, a triterpenoid library containing 66 compounds was established to evaluate their inhibitory effects against CYP 1A2, 2D6, 3A4, 2A6, 2B6, 2C9, and 2C19. CYP 1A2 was inhibited by most lanostane triterpenoids, indicating a strong affinity for these compounds. Among triterpenoids, compound **25** displayed a broad inhibitory effect against CYPs, except for CYP 3A4, 2D6, 2C9, and 2C19. Finally, compounds **6** and **25** exhibited interference with the metabolism of 16 drugs through the inhibition of CYPs *in vitro*. In silico molecular docking analyses for assaying the interaction between compound **25** and CYPs indicated that the hydrogen bonds formed between the hydroxyl groups and amino acid residues.

**Conclusion:**

*G*. *lucidum* displayed broad inhibitory effects on CYPs, with triterpenoids as the main bioactive constituents, which may induce potential drug-drug interaction. This information should be helpful for the rational use of *G*. *lucidum* in promoting human health.

## 1 Introduction

*Ganoderma lucidum* (*G*. *lucidum*) (Curtis) P. Karst, a traditional Chinese medicine and important resource of nutrient supplements, is the dried fruiting body of the polyporaceae fungus *Ganoderma lucidum* or *Ganoderma sinense*, which is widely distributed in Shandong, Anhui, Zhejiang, Jiangsu, Jiangxi, Fujian and other places in China (Ahmad, 2020). It was first recorded in the “Shennong Ben Cao Jing”, which has been used to improve health and treat many diseases in Asian countries for more than 2,000 years. It displayed various biological effects such as anti-tumor, anti-angiogenesis, anti-inflammatory, anti-herpes, anti-hypertension, anti-high cholesterol, anti-histamine, anti-complement, liver protection, and radiation protection (Ahmad, 2020; Ahmad et al., 2023; Chan et al., 2021; Su et al., 2020; Tong et al., 2023; Yu et al., 2021; Zhao et al., 2015; Zhen et al., 2023). Therefore, *G*. *lucidum* has been widely used in the prevention and treatment of various diseases, especially in liver protection, immune regulation, and clinical anti-tumor applications (Ahmad, 2020). So far, hundreds of compounds have been isolated and identified from *G*. *lucidum*, such as triterpenoids (Li et al., 2022; Lin et al., 2022), meroterpenoids (Cai et al., 2021), steroids (Zhang et al., 2008), polysaccharides (Liu et al., 2023), peptides (Zhao et al., 2015). Among them, water-soluble polysaccharides and triterpenoids are the main active components of *G*. *lucidum* (Carrieri et al., 2017; Xia et al., 2014). *G*. *lucidum* triterpenoids have attracted much attention due to their excellent pharmacological properties, and they are mostly highly oxidized lanostane type triterpenoids (Shiao, 2003).

Cytochrome P450 (CYP450) enzymes are a superfamily of heme proteins widely present in animals, plants, fungi, and bacteria. In mammals, CYP enzymes are widely expressed in almost all tissues, but their content is highest in the liver and small intestine (Manikandan and Nagini, 2018). In the human body, CYP enzymes are considered to be the superfamily of heme proteins and play a crucial role in the oxidative metabolism of endogenous and exogenous substances such as vitamins, cholesterol, prostaglandin, hormones, and clinical drugs (Tanaka, 1998). In addition, CYP enzymes distributed in liver and intestinal tissues are important drug metabolism enzymes, which play an important role in drug efficacy and detoxification (Lynch and Price, 2007; Tornio and Backman, 2018; Zhao et al., 2021). Among the 57 functional human CYP enzymes, only about 12 belong to the CYP 1, 2, and 3 families, responsible for the metabolic transformation of most foreign substances, including 70%–80% of clinically used drugs. The highest expression forms in the liver are CYP 3A4, 2C9, 2C8, 2E1, and 1A2, while 2A6, 2D6, 2B6, 2C19, and 3A5 are expressed at lower levels. CYP 2J2, 1A1, and 1B1 are mainly expressed outside the liver (Feng et al., 2022).

The interaction among many inducers, substrates, and inhibitors of CYP enzymes can change the metabolic behavior of drugs, leading to poor therapeutic effects of drugs or inducing adverse drug reactions (Machalz et al., 2021). Therefore, natural drugs and foods may regulate the metabolic function of CYP enzymes and affect the clinical drug metabolism process. *G*. *lucidum* related drugs and functional foods are widely used in healthcare and are often used in combination with various drugs (Swallah et al., 2023). Its effect on liver CYP enzymes activity and the resulting drug-drug interactions are still unclear (Wang et al., 2007). Therefore, it is of great significance for the safe and rational use of *G*. *lucidum* to study the effect of *G*. *lucidum* on CYP metabolic function, elaborate the active molecules, and evaluate the drug-drug interactions mediated by CYP enzymes.

Herein, on the basis of the combination of the CYP enzymes activity evaluation system, modern chromatography, and spectrum technology, we systematically studied the inhibition of *G*. *lucidum* on various subtypes of the CYP enzymes, clarified the main active substances of *G. lucidum* that intervene with CYP enzymes and their impact on clinical drug metabolism, and provided a reference for the rational use of *G. lucidum*.

## 2 Materials and methods

### 2.1 Reagents and enzymes

Chromatographic-grade methanol was purchased from Sigma–Aldrich. All other organic solvents were of chemical grade (Kermel Chemical Co. Ltd., Tianjin, China). Magnesium chloride, Monopotassium phosphate, Dipotassium phosphate, and dimethyl sulfoxide (DMSO) are from Maclean’s Reagent Co., Ltd. D-glucose-6-phosphate, glucose-6-phosphate dehydrogenase, dinucleotide phosphate disodium salt (NADP^+^) were purchased from Sigma-Aldrich (St. Louis, MO). Pooled human, rat and mouse liver microsomes were purchased from RILD Research Institute for Liver Disease Co. Ltd. (Shanghai, China). Drug standards such as midazolam, phenacetin, bupropion, diclofenac, mephenytoin, dextromethorphan, coumarin, sertraline, efavirenz, imatinib, simvastatin, diltiazem, glimepiride, glibenclamide and valsartan were purchased from Shanghai Yuanye Biotechnology Co., Ltd.

### 2.2 Apparatus

Analytical HPLC data were collected on a Waters e2695 instrument (Waters Corporation) equipped with a diode array detector (DAD). MS-100 constant temperature mixer (Hangzhou Aosheng). QTRAP 5500 LC/MS/MS and QTOF 550 LC/MS/MS (AB SCIEX).

### 2.3 Fungal material

Ethanolic extracts of the fruiting bodies of *Ganoderma lucidum* (Curtis) P. Karst. were purchased from Kingsci Biotechnology Co., Ltd. (China).

### 2.4 Inhibitory effect of dichloromethane extract from *Ganoderma lucidum* on CYP450 enzymes

The inhibitory effect of *G*. *lucidum* dichloromethane extract on seven subtypes of CYP enzymes (CYP1A2, 2A6, 2D6, 2B6, 2C9, 2C19, 3A4) was determined by co-incubation with human liver microsomes *in vitro*. At the same time, the species difference of *G*. *lucidum* dichloromethane extract on CYPs was determined by using different species of liver microsomes (human, mouse, rat).

The inhibitory effect of the dichloromethane extract of *G*. *lucidum* on seven subtypes of CYP enzymes (CYP1A2, 2A6, 2D6, 2B6, 2C9, 2C19, 3A4) was evaluated by specific substrate probe reaction (Supplementary Table S1). The total volume of the *in vitro* metabolic incubation system is 200 μL. The probe substrate concentration in the system is shown in Supplementary Table S1. 100 mM Tripotassium phosphate buffer solution (adjusted to pH 7.4 by K_2_HPO_4_/KH_2_PO_4_), contained NADPH generation system (composed of 1 unit/mL G-6-P-DH, 1 mM NADP, 10 mM G-6-P), HLM (0.5 mg/mL), 4 mM MgCl_2_ and 95% ethanol extract of *G*. *lucidum*, dichloromethane extract and extracted residual water layer (50 μg/mL). The reaction sample was preheated in a constant temperature mixer at 37°C. Then NADP^+^ was added to the system to initiate the reaction. The reaction time is 30 min, and 100 μL acetonitrile will be added on time to quickly terminate the reaction, followed by centrifugation at 4°C and 20,000 g for 20 min. The supernatant was analyzed using liquid chromatography tandem mass spectrometry (LC-MS/MS), and the activity of CYP enzymes was evaluated by the generation of probe metabolites. The mass spectrometry information of probe substrates is shown in Supplementary Table S3. Using liver microsome from three different species of human, rat and mouse, the species difference of inhibitory effect of dichloromethane extract of *G*. *lucidum* on CYP enzymes was determined in the above *in vitro* metabolic incubation system.

### 2.5 Interference of dichloromethane extract of *Ganoderma lucidum* on pharmacokinetics in clinical drugs

The interference of the dichloromethane extract of *G*. *lucidum* on the pharmacokinetics of phenacetin, bupropion, midazolam and diclofenac in rats were measured in order to further evaluate their inhibitory effects on CYP1A2, 2B6, 3A4 and 2C9.

SD rats (Male, weighing 220 g ± 20 g) were divided into a dichloromethane administration group and a control group with 6 rats in each group. Both groups were given probe substrate drugs by gavage. The drug administration group was given dichloromethane extract (500 mg/kg) by gavage 30 min before the substrate administration. 200 μL blood samples were taken from the rat jugular vein at 0.167, 0.333, 0.5, 0.75, 1, 2, 3, 4, 6, 8, 10, and 12 h after the administration of the probe substrate. After precipitation of proteins by with 600 μL acetonitrile via centrifugation (20000 g for 20 min) at 4°C at 3,500 rpm for 10 min. The supernatant was detected using liquid chromatography-tandem mass spectrometry (LC-MS/MS). The mass spectrometry information of the probe drug is shown in Supplementary Table S3. Animal experiments in this study were approved by the Animal Ethics Committee of the Dalian Medical University.

### 2.6 Evaluation of the inhibitory effect of triterpenoids on CYP enzymes

The isolated triterpenoids (**1**–**66**) were used to establish a compounds library. The inhibitory effects of 66 triterpenoids from *G*. *lucidum* on seven subtypes of CYP enzymes (CYP1A2, 2A6, 2D6, 2B6, 2C9, 2C19, 3A4) were evaluated followed the above-mentioned experimental procedures.

### 2.7 Investigation about the interference of main active triterpenoids on the metabolism of clinical drugs *in vitro*

The main active triterpenoids have significant inhibitory effects on the four subtypes CYP 1A2, 2B6, 2C9, and 3A4. For 16 clinical drugs metabolized by CYP enzymes, potential DDI studies were conducted on triterpenoids (**6**, **25**) of *G*. *lucidum*, and IC_50_ values were measured.

Clinical drugs: Phenacetin, melatonin, clozapine and riluzole are metabolized by CYP1A2. Bupropion, artemisinin, efavirenz and sertraline are metabolized by CYP2B6. Diclofenac, valsartan, glimepiride and glibenclamide are metabolized by CYP2C9. Midazolam, imatinib, simvastatin and diltiazem are metabolized by CYP3A4.

### 2.8 Mass spectrometry analysis and molecular network construction

10 mg of dichloromethane extract was dissolved in 1 mL of 50% acetonitrile-water solution, then filtrated and stored in 4°C. UPLC-Q/TOF-MS was implemented on a Sciex X500 system combined with ExionLC + PDA system (Sciex, Massachusetts, United States). The detailed conditions of HPLC and UPLC-Q/TOF-MS were shown in Supplementary Table S3. Molecular network of dichloromethane extract was constructed based on the operation guidelines in GNPS (https://gnps.ucsd.edu/ProteoSAFe/static/gnps-splash.jsp).

### 2.9 Docking analysis

The Cytochrome P450 1A2 (CYP1A2, PDB ID: 2HI4), 2B6 (CYP2B6, PDB ID: 5UDA), 3A4 (CYP3A4, PDB ID: 4D7D), 2C9 (CYP2C9, PDB ID: 4NZ2) and 2C19 (CYP2C19, PDB ID: 4GQS) retrieved from the protein data bank was utilized to conduct the docking study using Discovery Studio 2016. The receptor was prepared by protein preparation wizard, while the ligands were prepared by the ligand preparation wizard. The active site was determined based on its original co-crystallization. Docking was commenced by LibDock software found in the Discovery Studio 2016 package.

### 2.10 Molecular dynamics

In the current study, we performed five molecular dynamic simulation experiments to support our concept of design. All complexes were solvated with solvation package to add water molecules to the cubic simulation boxes. Energy minimization was achieved by employing the steepest descent minimization algorithm with a maximum of 1,000 steps and a constant number of particles, pressure, and temperature for 200 ps. A constant temperature of 300 K and constant pressure of 1 atm were maintained through the entire MD simulation. The entire MD simulation experiments were conducted using Discovery Studio 2016 standard dynamics cascade package at charmm27 force field. The root mean square deviation (RMSD) and root mean square fluctuation (RMSF) were calculated from the generated trajectories of the MD simulations as well as the distances of the formed hydrogen bonds between receptor and ligand.

### 2.11 Statistical approaches

All of the experiments were repeated at least three times with the data presented as means ± standard error (SE) using one-way ANOVA to determine significant differences of multi groups. Unpaired Student’s t-test was used to assess differences between two groups. Results were considered to be statistically significant differences at *P* < 0.05. PCA was performed in R version 3.3.3. and plotted by https://www.bioinformatics.com.cn.

## 3 Results

### 3.1 The extracts of *Ganoderma lucidum* inhibit CYPs in different species

**FIGURE 1:**
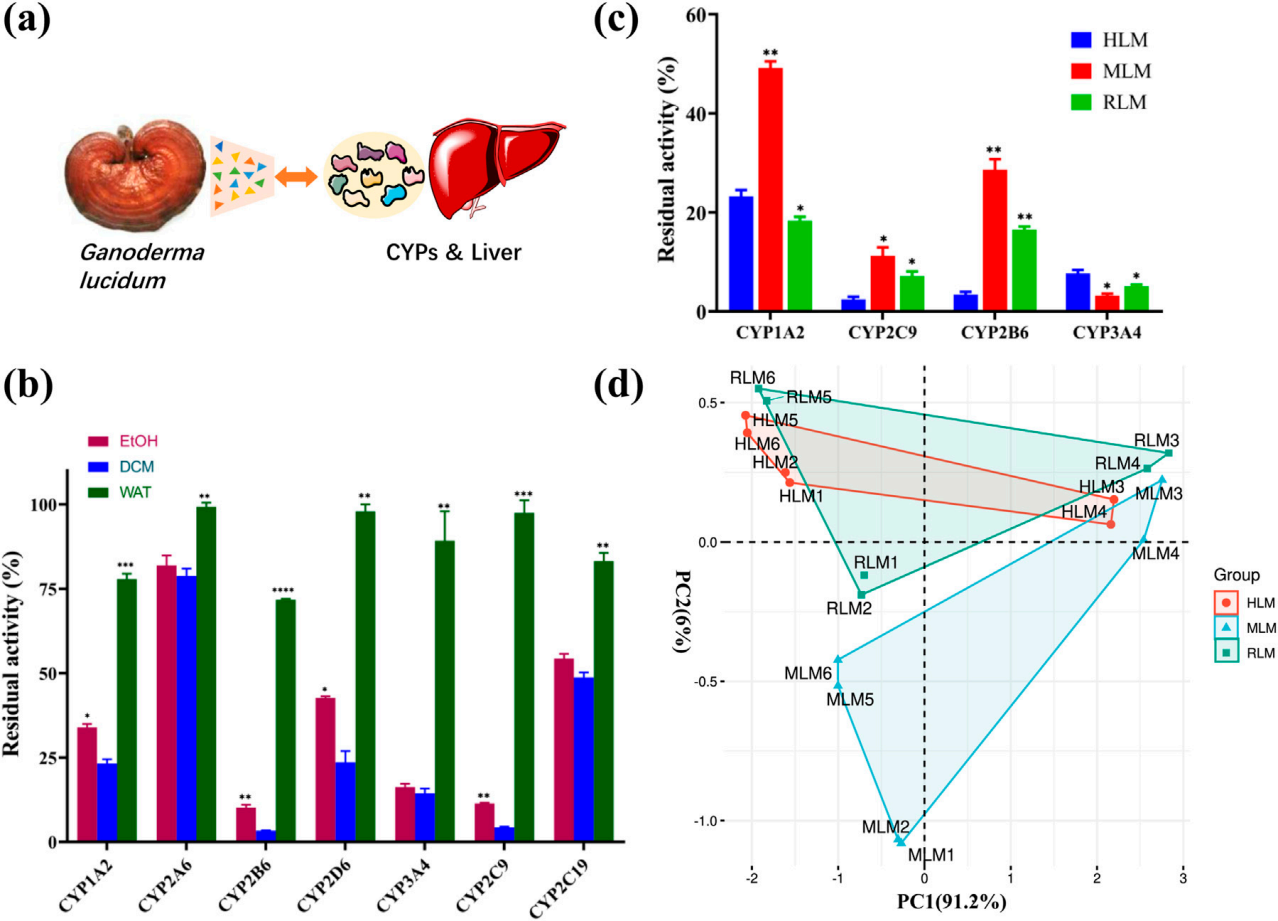
The extracts of *Ganoderma lucidum* inhibit CYPs from different species. **(A)** Illustration about the study of *Ganoderma lucidum* against the enzymatic function of CYPs in liver; **(B)** The inhibitory effect of different extracts of *Ganoderma lucidum* against various CYPs; **(C)** The inhibitory effect of dichloromethane extract against various CYPs from different species; **(D)** The principal component analysis (PCA) about the inhibitory effect of dichloromethane extract against various CYPs from different species (6 samples). Human CYP1A2, CYP2C9, CYP2B6 and CYP3A4 represent the corresponding isoenzymes in rat and mouse, respectively. All of the experiments were repeated three times (n = 3). HLM (human liver microsomes), MLM (mouse liver microsomes), RLM (rat liver microsomes), EtOH (ethanol extract), DCM (Dichloromethane extract), WAT (Water extract).

The present study was performed to explore the chemical and biological mechanism of *G*. *lucidum* against CYPs (Figure 1A). Using special substrates of seven CYPs, respectively, the ethanol extract, dichloromethane extract, water extract of the fruiting body of *G*. *lucidum* were evaluated for their inhibitory effects on seven CYPs from human hepatic microsomes. As shown in Figure 1B, the water extract displayed weak inhibitory effect. However, the dichloromethane extract displayed the strongest inhibitory effect on CYP 1A2, 2A6, 2B6, 2D6, 3A4, and 2C9, together with a moderate inhibitory effect on 2C19 (Figure 1B).

On the other hand, the inhibitory effects of different extracts of *G. lucidum* against CYPs (CYP 2C9, CYP 3A4, CYP 1A2, CYP 2B6) from different species (human, mouse, rat) were evaluated to reveal the species differences about the inhibitory effects of *G*. *lucidum* (Supplementary Figure S1). It was obvious that the dichloromethane extract could inhibit CYPs from three species, which was recognized as the strongest active extract (Figure 1C). In addition, principal component analysis (PCA) has been performed for the inhibitory effects of different extracts on CYPs from different species. As shown in Figure 1D, more cluster similarity was observed for the CYPs inhibition on human and rat species, which suggested that the rat could be a suitable animal model for the study of CYPs inhibition by *G. lucidum*.

### 3.2 The extract of *Ganoderma lucidum* interfered with the pharmacokinetic of clinical drugs by inhibiting CYPs

**FIGURE 2:**
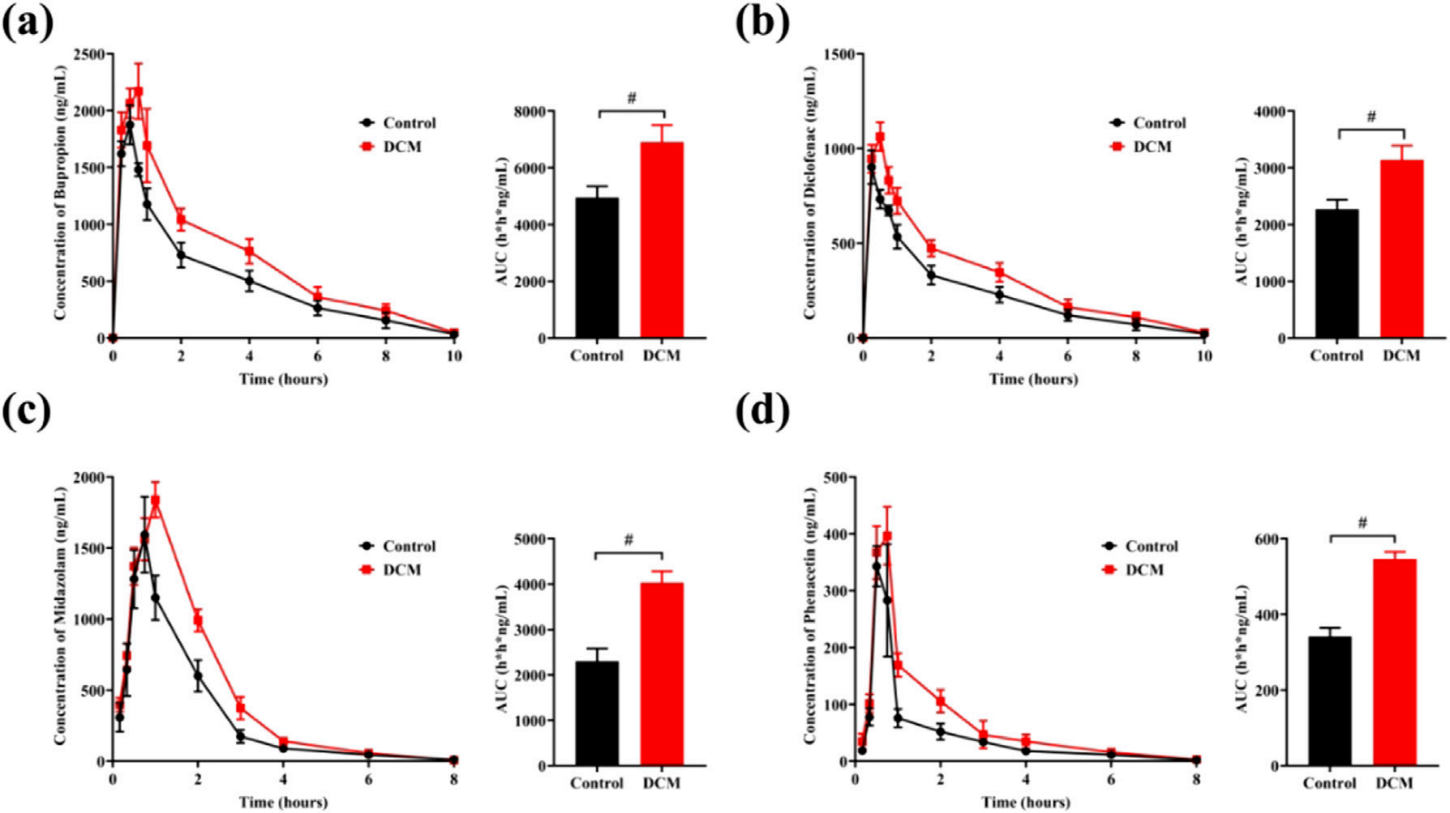
The dichloromethane extract of *Ganoderma lucidum* interfered with the metabolic process of clinical drugs (n = 6). **(A)** Drug concentration-time curve and AUC of bupropion; **(B)** Drug concentration-time curve and AUC of diclofenac; **(C)** Drug concentration-time curve and AUC of midazolam; **(D)** Drug concentration-time curve and AUC of phenacetin.

It was known that liver mediated the metabolism of most drugs, and CYPs played an important role in the drugs metabolism. Herein, the interference of *G. lucidum* on the *in vivo* metabolism of some typical drugs was investigated. The average blood drug concentration time distribution curves and the area under the curve (n = 6) are shown in Figure 2. The experimental results showed that the dichloromethane extract of *G*. *lucidum* had a significant impact on the pharmacokinetics of phenacetin, diclofenac, midazolam, and bupropion in rats. The area under the curve of the *G*. *lucidum* administration group was significantly higher than that of the control group, indicating that the dichloromethane extract of *G. lucidum* could significantly inhibit the metabolic activity of rat liver CYP enzymes (especial CYP2B6, CYP2C9, CYP3A4, and CYP1A2), and then affect the related drug metabolism process.

As shown in Table 1, the area under the blood drug concentration time curve (AUC), peak time (T_max_), and maximum blood drug concentration (C_max_) were calculated using Phoenix software. It was obvious that administration of *G*. *lucidum* could increase the values of AUC, T_max_, and C_max_, indicating the bioavailability increasing of four drugs.

**TABLE 1:** Pharmacokinetic parameters of four clinical drugs in rat with the presence of dichloromethane extract (DCM) of *Ganoderma lucidum*. Phenacetin (PHE), diclofenac (Dic), midazolam (MDZ), bupropion (Bup).

	PHE	PHE + DCM	*P*-value	Dic	Dic + DCM	*P*-value
T_max_ (h)	0.625 ± 0.137	0.667 ± 0.129	*	0.292 ± 0.102	0.458± 0.102	*
C_max_ (ng/mL)	364.233 ± 38.0877	407.667 ± 47.605	*	912.0 ± 68.118	1,068.667 ± 74.207	**
AUC (0-t) (h*ng/mL)	341.958 ± 22.570	546.004 ± 19.325	#	2,268.688 ± 167.841	3,139.708 ±251.506	#
AUC (0-∞) (h*ng/mL)	351.720 ± 22.602	564.694 ± 27.546	#	2,353.482 ± 199.217	3,250.257 ± 237.272	#
Vz_F (mL/kg)	31,514.554 ± 6,298.860	18,399.085 ± 5,435.352	**	12,244.920 ± 1707.693	8,790.851 ± 2025.109	**
Cl_F_ (mL/h/kg)	14,264.696 ± 912.942	8,872.347 ± 443.073	#	4,276.363 ± 388.068	3,090.516 ± 227.704	#
AUM (h*ng/mL)	692.744 ± 85.4782	1,155.668 ± 163.174	***	7,332.465 ± 1,560.101	10,429.580 ± 1,102.722	**

	MDZ	MDZ + DCM	*P*-value	Bup	Bup + DCM	*P*-value
T_max_ (h)	0.667 ± 0.129	0.958 ± 0.102	**	0.417 ± 0.129	0.750 ± 0.158	***
C_max_ (ng/mL)	1,629.417 ± 239.536	1849.667 ± 108.188	*	1888.000 ± 154.066	2,265.267 ± 132.510	***
AUC (0-t) (h*ng/mL)	2,303.808 ± 279.727	4,031.277 ± 251.753	#	4,951.746 ± 392.684	6,909.827 ± 593.183	#
AUC (0-∞) (h*ng/mL)	2,344.588 ± 273.272	4,046.162 ± 259.048	#	5,093.624 ± 455.627	7,098.891 ± 593.496	***
Vz_F (mL/kg)	8,995.776 ± 2,395.823	3,154.289 ± 777.285	***	10,405.594 ± 1,602.189	7,445.840 ± 1,541.101	***
Cl_F_ (mL/h/kg)	4,313.217 ± 497.661	2,479.719 ± 154.593	#	3,954.386 ± 375.228	2,834.313 ± 244.118	***
AUM (h*ng/mL)	4,197.381 ± 670.735	7,430.947 ± 802.923	#	15,378.075 ± 3,089.500	22,117.336 ± 2,737.892	***

*P < 0.05, **P < 0.01, ***P < 0.001, ^#^ *P* < 0.0001.

### 3.3 Lanostane type triterpenoids are the major chemical constituents of dichloromethane extract

**FIGURE 3:**
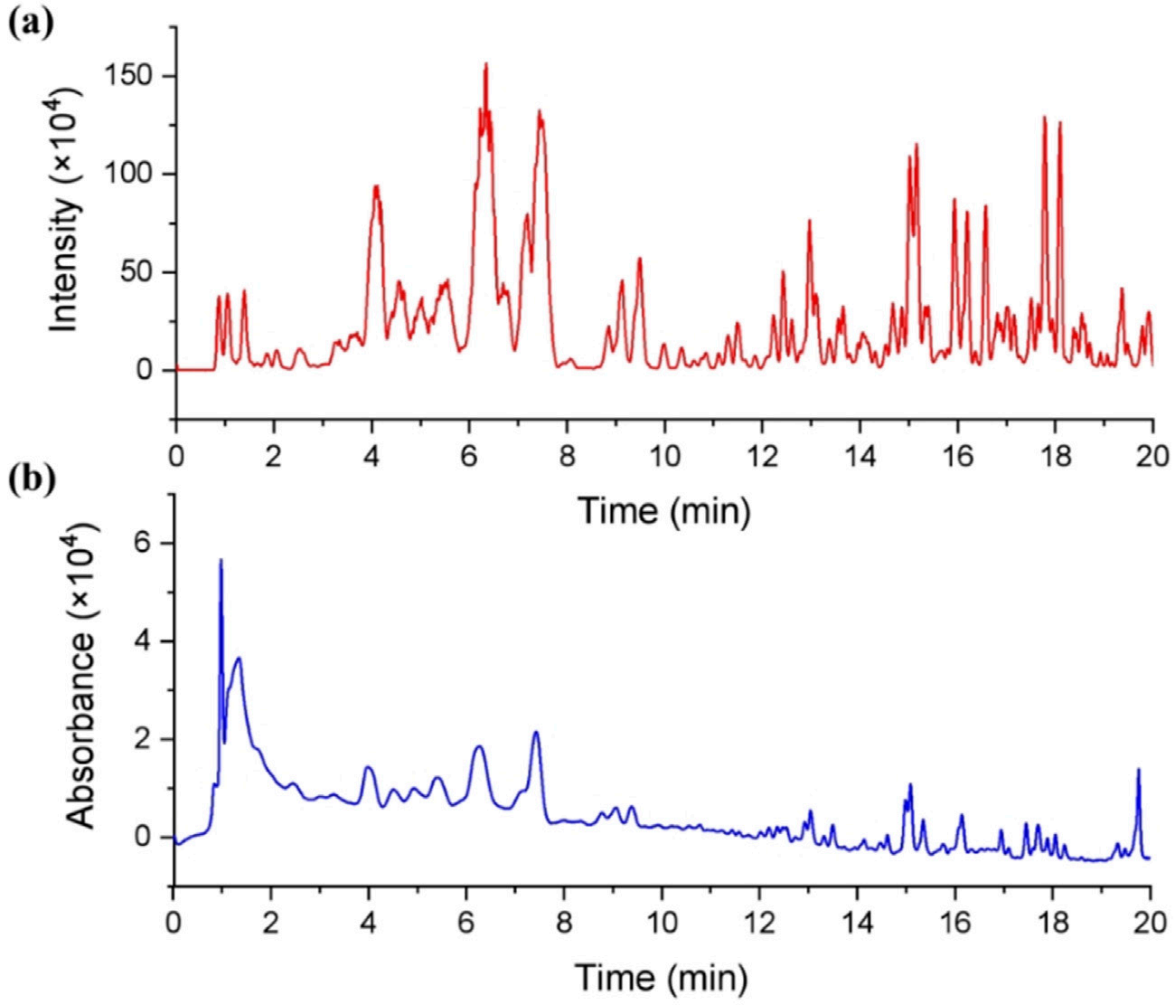
Chromatographic characteristics of the dichloromethane extract of *Ganoderma lucidum*. **(A)** Total ion chromatogram of the dichloromethane extract of *Ganoderma lucidum* in ESI positive mode; **(B)** HPLC chromatogram of the dichloromethane extract of *Ganoderma lucidum* (190–700 nm).

**FIGURE 4:**
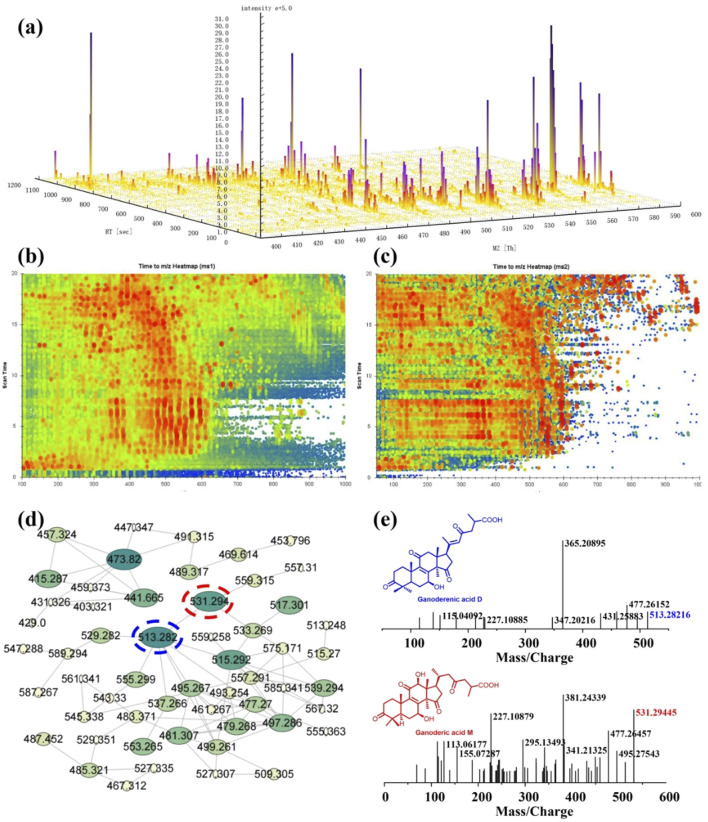
Chemical constituent analysis of the dichloromethane extract of *Ganoderma lucidum* using LC-MS. **(A)** The 3D view of the major parent ions; **(B)** and **(C)** Time to *m/z* heatmap of MS1 **(B)** and MS2 **(C)**; **(D)** The molecular network of the dichloromethane extract; **(E)** The MS spectrum of typical triterpenoids including ganoderenic acid D ([M + H]^+^ *m/z* 513.28216) and ganoderic acid M ([M + H]^+^ *m/z* 531.29445).

As the significant inhibitory effect on CYPs, the chemical constituents of dichloromethane extract of *G. lucidum* are desired to be revealed. Therefore, LC-MS is used to identify the constituents of DCM. From the ESI positive ion mode ion flow diagram, it can be seen that there were continuous ion response peaks from beginning to end, especially multiple strong response signals at 3.8–8.0 min and 15.0–18.3 min (Figure 3A), which is consistent with the DAD chromatographic characteristics of dichloromethane extract (Figure 3B) and indicates that the dichloromethane components contain rich material compositions. From the 3D and 2D thermograms (Figures 4A, B) of the distribution of molecular ion peak *m/z*, the molecular ion peaks of the components, eluded between 3.8–15 min, are concentrated between 500–600 *m/z*, while the molecular ion peaks of compounds between 15–18 min are mainly concentrated between 400–500 *m/z*, indicating that the chemical composition of dichloromethane extract may mainly be divided into two categories. As shown in Figure 4C, the MS2 characteristics of the two types of compounds are highly similar, indicating that these two types of components may have the same or similar mother nucleus structures.

Molecular network (MN) technology is a highly clustered mass spectrometry analysis technique that can deeply explore the diversity and derivative relationships of components based on the similarity relationship of secondary mass spectrometry, provide useful references for elucidating mixture components and guide separation. Therefore, we uploaded the mass spectrometry data of dichloromethane extract to the GNPS mass spectrometry library and further utilized the MN module to conduct molecular network analysis of dichloromethane extract (Figure 4D). The results indicate that there are multiple quasi-molecular ions with a mass to nucleus ratio of 400–600 *m/z* in dichloromethane extract with structural derivation relationships. A type of substance with lower molecular weight is characterized by a quasi-molecular ion with a mass to nucleus ratio of 473.820 as the key node, but the secondary mass spectrometry of this compound is not included in the mass spectrometry library. The key nodes for substances with higher molecular weights are the mass to nucleus ratios of 531.294, 513.282, and 515.292. Through database comparison and identification, the compound corresponding to the molecular ion peak of 531.294 is ganoderic acid M ([M + H]^+^ *m/z* 531.29445), while the compound corresponding to the molecular ion peak of 513.282 is ganoderic acid D ([M + H]^+^ *m/z* 513.28216). Their secondary mass spectrometry fragmentation characteristics are shown in Figure 4E. This is consistent with the chemical composition previously reported by *G. lucidum* (Gong et al., 2019), indicating that the main chemical component type of DCM component is triterpenoid. All data provided a reference and basis for subsequent separation and structural identification experiments.

### 3.4 Triterpenoids from *Ganoderma lucidum* inhibit human CYPs

**FIGURE 5:**
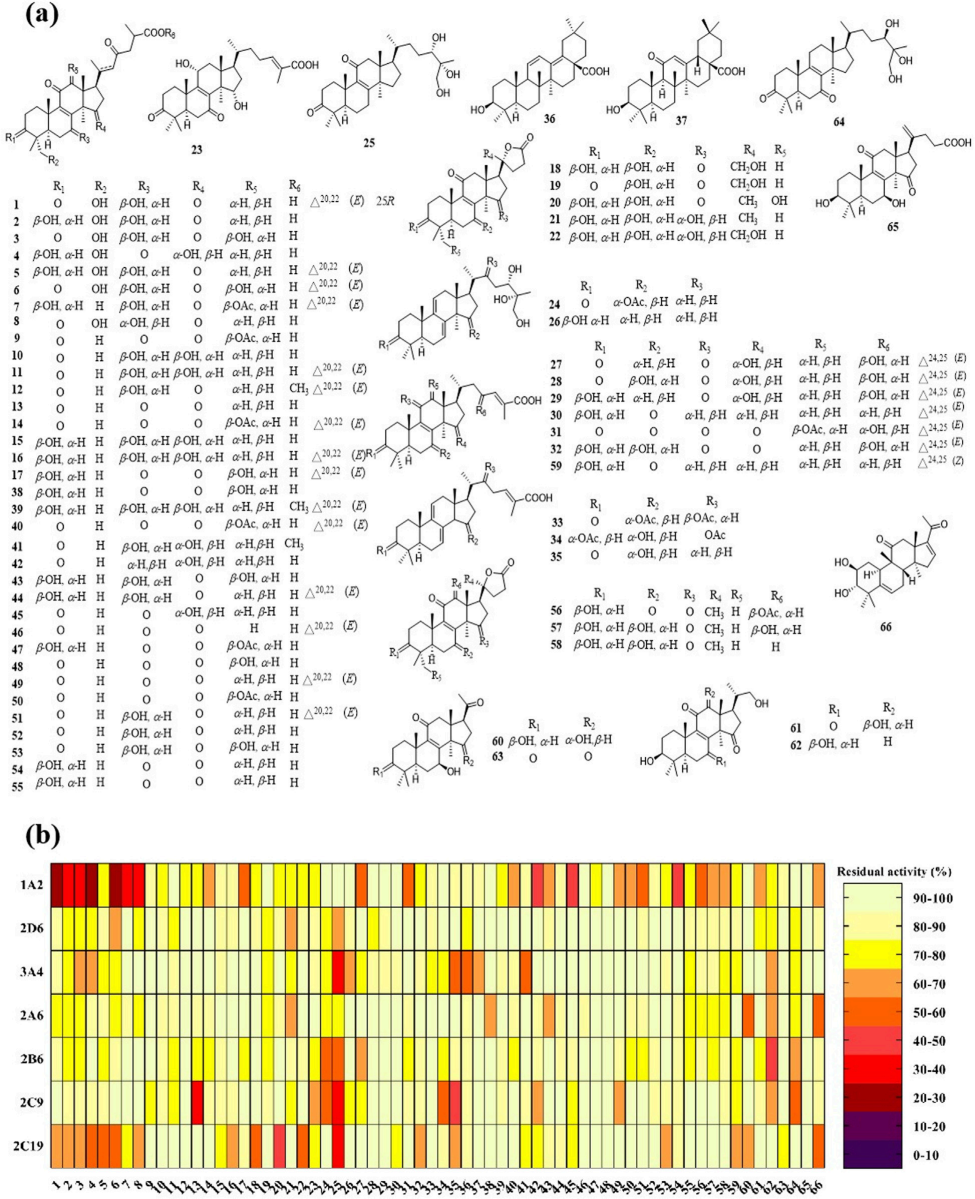
Triterpenoids of *Ganoderma lucidum* and their inhibitory effect on CYPs. **(A)** The chemical structures of triterpenoids **1**–**66**; **(B)** The inhibitory effect of triterpenoids against CYP 1A2, 2D6, 3A4, 2A6, 2B6, 2C9, and 2C19.

On the basis of our previous large-scale isolation and identification of chemical constituents for the mushroom of *G*. *lucidum*, serious triterpenoids were obtained from *G*. *lucidum*, which established a compound library containing 66 triterpenoids, most of which were lanostane type triterpenoids (Figure 5A).

**FIGURE 6:**
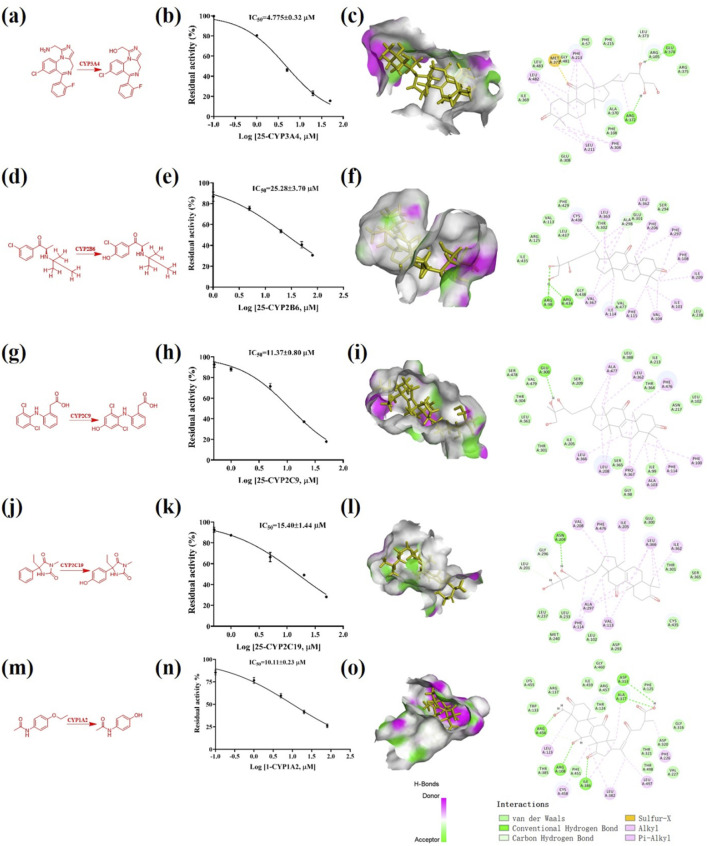
Triterpenoid **25** inhibited multiple CYPs. **(A)** The enzymatic reaction scheme about the activity assay of CYP 3A4 using midazolam as the substrate; **(B)** The IC_50_ determination of **25** against CYP 3A4; **(C)** In silico docking analysis about **25** and CYP 3A4; **(D)** The enzymatic reaction scheme about the activity assay of CYP 2B6 using bupropion as the substrate; **(E)** The IC_50_ determination of **25** against CYP 2B6; **(F)** In silico docking analysis about **25** and CYP 2B6; **(G)** The enzymatic reaction scheme about the activity assay of CYP 2C9 using diclofenac as the substrate; **(H)** The IC_50_ determination of **25** against CYP 2C9; **(I)** In silico docking analysis about **25** and CYP 2C9; **(J)** The enzymatic reaction scheme about the activity assay of CYP 2C19 using mephenytoin as the substrate; **(K)** The IC_50_ determination of **25** against CYP 2C19; **(L)** In silico docking analysis about **25** and CYP 2C19; **(M)** The enzymatic reaction scheme about the activity assay of CYP 1A2 using mephenytoin as the substrate; **(N)** The IC_50_ determination of **1** against CYP 1A2; **(O)** In silico docking analysis about **1** and CYP 1A2.

Using the *in vitro* bioassay technique, all of the triterpenoids **1**–**66** were evaluated for their inhibitory effect against CYP 1A2, 2D6, 3A4, 2A6, 2B6, 2C9, and 2C19 (Figure 5B). As shown in the heat map, CYP 1A2 was inhibited by the most triterpenoids, which meant a good affinity for these lanostane type triterpenoids. On the other hand, the ganoderma triterpenoids displayed a weak inhibitory effect on CYP 2A6 and 2D6. Among different triterpenoids, compound **25** displayed a broad inhibitory effect against CYPs. The IC_50_ values of **25** against CYP 3A4, 2B6, 2C9, and 2C19 were further determined to be 4.78, 25.28, 11.37, and 15.40 μM, respectively (Figure 6). In silico docking analysis was also performed between **25** and CYP 3A4, 2B6, 2C9, and 2C19, which not only indicated the binding between **25** and CYPs, but also revealed the potential interaction between **25** and CYPs, especially the hydrogen bonds formed between the hydroxyl groups located at the side chain and amino acid residues.

As shown in the heat map, most of the triterpenoids could inhibit CYP 1A2, suggesting the widely affinity between CYP 1A2 and lanostane type triterpenoids. There were 14 triterpenoids were determined their detailed inhibitory effect on CYP 1A2, and their IC_50_ values ranged from 9.6 to 38.56 μM, among which triterpenoid **6** displayed the strongest inhibitory effect (Table 2). As shown in Figure 5, all of these 14 triterpenoids belonged to lanostane type triterpenoid, possessing 30 carbons in their chemical skeleton. They were all highly oxygenated, possessing unsaturated ketones and carboxyl group at the side chain.

**TABLE 2:** The IC_50_ values of typical triterpenoids against CYP 1A2.

Compounds	IC_50_ (μM)	Compounds	IC_50_ (μM)
**1**	10.11 ± 0.23	**17**	21.78 ± 1.15
**2**	16.03 ± 4.05	**31**	29.02 ± 6.91
**3**	13.96 ± 1.73	**42**	21.40 ± 5.21
**4**	11.60 ± 1.02	**45**	18.25 ± 1.33
**6**	9.62 ± 2.03	**51**	38.56 ± 9.40
**7**	12.80 ± 1.47	**54**	24.67 ± 3.38
**8**	11.31 ± 0.31	**56**	34.19 ± 6.90

**FIGURE 7:**
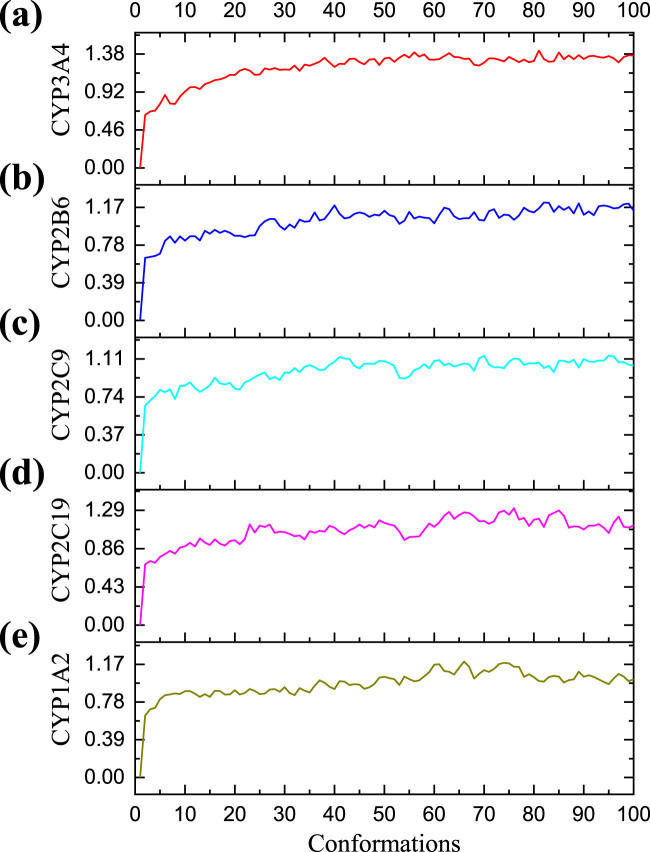
The RMSD of five molecular dynamic (MD) simulation experiments. **(A)** The RMSD of Cytochrome P450 3A4 (CYP3A4)-**25** protein ligand complex in MD simulation; **(B)** The RMSD of Cytochrome P450 2B6 (CYP2B6)-**25** protein ligand complex in MD simulation; **(C)** The RMSD of Cytochrome P450 2C9 (CYP2C9)-**25** protein ligand complex in MD simulation; **(D)** The RMSD of Cytochrome P450 2C19 (CYP2C19)-**25** protein ligand complex in MD simulation; **(E)** The RMSD of Cytochrome P450 1A2 (CYP1A2)-**1** protein ligand complex in MD simulation. The horizontal axis represents the frame number of simulation, and the vertical axis represents the RMSD value of each conformation.

The optimal conformations for the combination of CYP3A4, CYP2B6, CYP2C9, and CYP2C19 with compound **25** generated in the early docking experiments, as well as the optimal conformation for the combination of CYP1A2 with compound **1**, were solvated using a periodic boundary model. In order to investigate and analyze the structural stabilities of protein-ligand complexes in simulated physiological environments, water molecules (17,653), solids (67), and chlorides (47) were sequentially added to the ligand-receptor complex. Under CHARMM force field with simulated time of 200 ps, the RMSD values of the five complexes during MD simulation are shown in Figure 7. The complexes in the five systems reached stability after MD simulation at 200 ps. The RMSD value of CYP3A4-**25** complex mainly fluctuates between 0.64 and 1.42, with a mean RMSD of 1.21; The RMSD value of CYP2B6-**25** complex mainly fluctuates between 0.65 and 1.22, with a mean RMSD of 1.03; The RMSD value of CYP2C9-**25** complex mainly fluctuates between 0.66 and 1.14, with a mean RMSD of 0.98; The RMSD value of CYP2C19-**25** complex mainly fluctuates between 0.68 and 1.29, with a mean RMSD of 1.06; The RMSD value of CYP1A2-**1** complex mainly fluctuates between 0.64 and 1.20, with a mean RMSD of 0.97. The RMSD fluctuation values of the five complexes are all within a reasonable range, indicating that the structure of the complexes in the system is in an equilibrium state after simulation. Ligands **25** and **1** can bind to the five proteins respectively, inhibiting their enzymatic reaction performance.

**FIGURE 8:**
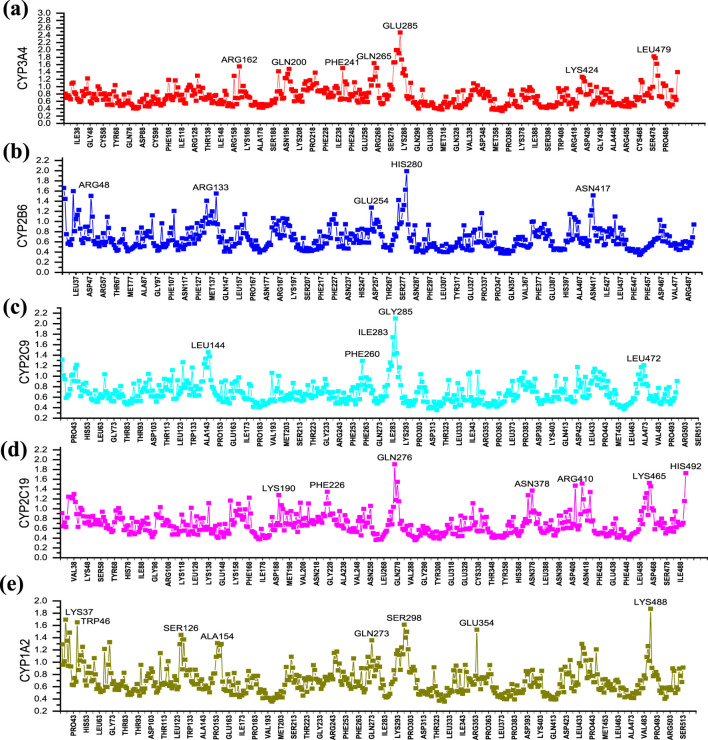
The RMSF of five dynamic simulation experiments. **(A)** Cytochrome P450 3A4 (CYP3A4) with ligand **25**; **(B)** Cytochrome P450 2B6 (CYP2B6) with ligand **25**; **(C)** Cytochrome P450 2C9 (CYP2C9) with ligand **25**; **(D)** Cytochrome P450 2C19 (CYP2C19) with ligand **25**; **(E)** Cytochrome P450 1A2 (CYP1A2) with ligand **1**. The horizontal axis lists the amino acid residues of the protein, and the vertical axis represents the RMSF value of each residue.

In order to analyze the fluctuations of various amino acids in the complex during the MD simulation process, the RMSF values of all amino acids during the simulation process were calculated (Figures 8A–E). As shown in Figure 8A, the CYP3A4-**25** complex exhibits significant fluctuations around amino acids ARG162, GLN200, PHE241, GLN265, GLU285, LYS424, and LEU479; CYP2B6-**25** complex exhibits significant fluctuations around amino acids ARG48, ARG133, GLU254, HIS280, and ASN417; CYP2C9-**25** complex exhibits significant fluctuations around amino acids LEU144, GLY285, and LEU472; CYP2C19-**25** complex exhibits significant fluctuations around amino acids GLN276, ASN378, ARG410, LYS465, and HIS492; while the CYP1A2-**1** complex exhibits significant fluctuations around amino acids LYS37, TRP46, SER126, ALA154, GLN273, SER298, GLU354, and LYS488. Overall, the fluctuation of amino acids in the five complexes is relatively small, indicating that the complexes have good stability.

### 3.5 Active triterpenoids inhibit the *in vitro* metabolism of clinical drugs mediated by CYPs

Based on the aforementioned study, lanostane type triterpenoids were identified as the major active constituents of DCM against CYPs, especially ganodrol C (**25**), ganoderica F (**6**). Therefore, the interference of triterpenoids with the metabolism of clinical drugs should be explored *in vitro* or *in vivo*. As shown in Table 3, midazolam (CYP 3A4), simvastatin (CYP 3A4), diltiazem (CYP 3A4), and imatinib (CYP 3A4), bupropion (CYP 2B6), artemisinin (CYP 2B6), efavirenz (CYP 2B6), sertraline (CYP 2B6), diclofenac (CYP 2C9), valsartan (CYP 2C9), glimepiride (CYP 2C9), glibenclamide (CYP 2C9) were previously known to be metabolized by special CYP enzymes, and they were co-incubated with ganodrol C (**25**) to detect the production of corresponding metabolites. As a result, ganodrol C could inhibit the *in vitro* metabolism of these drugs mediated by special CYPs with IC_50_ values ranging from 4.50 μM to 25.28 μM. Therefore, ganodrol C displayed broad interference with drug metabolism mediated by CYP 3A4, 2B6, and 2C9.

**TABLE 3:** The inhibitory effect of ganodrol C (**25**) against the metabolism of various clinical drugs mediated by CYP 3A4, 2B6, and 2C9.

CYP subtype	Drugs	IC_50_ (μM)
CYP3A4	Midazolam	4.78 ± 0.32
	Simvastatin	4.50 ± 0.48
	Diltiazem	5.12 ± 0.56
	Imatinib	7.57 ± 0.20
CYP2B6	Bupropion	25.28 ± 3.70
	Artemisinin	21.53 ± 3.44
	Efavirenz	24.31 ± 3.05
	Sertraline	25.06 ± 2.86
CYP2C9	Diclofenac	11.37 ± 080
	Valsartan	14.26 ± 1.14
	Glimepiride	12.31 ± 0.43
	Glibenclamide	10.26 ± 0.12

On the other hand, phenacetin, riluzole, clozapine, and melatonin were reported to undergo a metabolism process mediated by CYP 1A2. Herein, ganoderica F (**6**), a significant inhibitor of CYP 1A2, was co-incubated with the aforementioned drugs to evaluate its potential interference with metabolism *in vitro*. As shown in Table 4, ganoderica F could inhibit the metabolism of four drugs mediated by CYP 1A2, with IC_50_ values ranging from 9.62 μM to 18.96 μM.

**TABLE 4:** The inhibitory effect of ganoderica F (**6**) against the metabolism of various clinical drugs mediated by CYP 1A2.

CYP subtype	Drugs	IC_50_ (μM)
CYP1A2	Phenacetin	9.62 ± 2.03
	Riluzole	14.79 ± 2.15
	Clozapine	12.93 ± 1.11
	Melatonin	18.96 ± 1.68

## 4 Discussion

In previous study, polysaccharides from *G. lucidum* have been shown to exhibit inhibitory activity against CYP 2E1, CYP 1A2 and CYP 3A in hepatic microsomes, which indicated the potential interference with pharmacokinetics of drugs, even drug-herb interaction (Wang et al., 2007). However, the bioactive constituents of *G*. *lucidum* against CYPs, along with their mechanisms and drug-herb interaction, are still not clear. Therefore, the chemical and biological mechanism of *G. lucidum* against CYPs was examined in this study. The results showed that the dichloromethane extract of *G. lucidum* was the most effective CYPs inhibitor, and the rat was identified as the animal model for studying the inhibitory effect of *G. lucidum* on CYPs.

Based on the results of *in vitro* inhibition screening, we further verified the inhibitory effect of *G. lucidum* extract on CYPs through *in vivo* drug metabolism experiments. A single dose pharmacokinetic study was conducted in a rat model to explore the interference of the dichloromethane extract of *G*. *lucidum* on the metabolism of four clinical drugs *in vivo*. In previous studies, bupropion, diclofenac, midazolam, and phenacetin were reported as the special substrate of CYP 2B6, CYP 2C9, CYP 3A4, and CYP 1A2, respectively, which meant the special metabolism process *in vivo* (Pillai et al., 2013). So, the combined administration of *G*. *lucidum* and 4 drugs has been performed in rats, respectively, along with the pharmacokinetic study of 4 drugs in comparison with the individually administered rats by 4 drugs. As a result, the dichloromethane extract of *G. lucidum* could inhibit the CYPs of rats, displaying interference with the metabolism of drugs mediated by corresponding CYPs, as well as the potential drug-drug interactions based on the metabolism.

As above mentioned, the dichloromethane extract of *G*. *lucidum* displayed a significant inhibitory effect on CYPs, along with the interference with drug metabolism. Thus, the bioactive constituents of dichloromethane extract attracted our interest. Utilizing the UPLC-Q-TOF-MS, the major constituents of dichloromethane extract were detected and identified in preliminary analyses. As a result, the major constituents of dichloromethane extract were proposed to be lanostane type triterpenoids.

In the previous studies of our team, a lot of triterpenoids were isolated from *G. lucidum*, which constructed a compounds library (Li et al., 2022; Li et al., 2023; Lin et al., 2022; Wei et al., 2018; Zhao et al., 2016). Herein, 66 triterpenoids were evaluated for their inhibitory effects on CYPs in this study. CYP 1A2 was inhibited by most lanostane triterpenoids, indicating a strong affinity for these compounds. Compound **25** showed a broad inhibitory effect against CYPs. The interaction between active triterpenoid **25** and CYPs was examined by *in silico* molecular docking, which indicated hydrogen bonds formed between the hydroxyl groups and amino acid residues.

It was known that CYPs participated in the metabolism of most drugs, playing an important role in their bioavailability, bioactivity, and even toxicity (Manikandan and Nagini, 2018). Thus, the inhibitors of CYPs also can be proposed to interfere with drugs *in vivo*, inducing drug-drug interaction. Herein, 16 drugs were used as the substrates of four CYPs (3A4, 2B6, 2C9, 1A2), respectively to evaluate the inhibitory effect of ganoderica F (**6**) and ganodrol C (**25**) *in vitro*, along with the prediction of drug-drug interactions. It is worth noting that ganoderica F (**6**) and ganodrol C (**25**), as inhibitors of CYPs, could inhibit the metabolism of clinical drugs, displaying the potential for drug-drug interactions.

The Pharmacopoeia of the People’s Republic of China (Chinese Pharmacopoeia 2000 edition) includes *G. lucidum*, which is recognized for its clear medicinal value in China. In clinical studies, several investigations have revealed the synergistic effects of the combination of *G. lucidum* and commonly used drugs, which may be induced by interference with liver metabolism of these drugs (Lam et al., 2020; Suárez-Arroyo et al., 2016). Currently, *G. lucidum* is utilized not only as a therapeutic agent for the clinical prevention and treatment of diseases but also as a health food for healthcare. In addition, a dosage of dichloromethane extract of *G. lucidum* (500 mg/kg, rat) was administered by oral gavage in the study, along with the dosage guidelines (6–12 g/day) outlined in the 2020 edition of the Pharmacopoeia of the People’s Republic of China, which can provide an effective reference for the rational clinical use or tonic diet of *G. lucidum*.

## 5 Conclusion

*G*. *lucidum* was widely used as traditional medicine and functional food for human health. In consideration of its various biological effects, *G*. *lucidum* was usually used in combination with other drugs in clinic. In this study, *G. lucidum* was found to have a broad inhibitory effect on various CYP enzymes *in vitro* and *in vivo*. By utilizing chromatographic technique and spectroscopic method, a triterpenoid library was established from *G*. *lucidum*, which displayed inhibitory effects against various CYPs, especially CYP 1A2, 3A4, 2B6, and 2C19. Among these CYPs, CYP 1A2 was widely inhibited by most of the triterpenoids, especially the lanostane type triterpenoids. In addition, ganodrol C (**25**) could inhibit multiple CYPs and interfere with the metabolism of clinical drugs mediated by CYPs. Therefore, *G*. *lucidum* and its bioactive constituents, lanostane triterpenoids, were explored for their inhibitory effects on CYPs, as well as their interference with the metabolic processes of different drugs, indicating the potential for drug-drug interactions. This information should be helpful for the rational use of *G. lucidum* in promoting human health.

## Data Availability

The raw data supporting the conclusions of this article will be made available by the authors, without undue reservation.
