# Erratum: Triterpenoids from *Ganoderma lucidum* inhibit cytochrome P450 enzymes interfering with the metabolic process of specific clinical drugs

**DOI:** 10.3389/fphar.2024.1539089

**Published:** 2024-12-19

**Authors:** Frontiers Production Office

**Affiliations:** Frontiers Media SA, Lausanne, Switzerland

**Keywords:** *Ganoderma lucidum*, functional food, CYP450s, lanostrane triterpenoid, drug-drug interactions

Due to a production error, the funders “National Natural Science Foundation of China (82374297), Dalian Science and Technology Leading Talents Project (2020RJ09), Dalian Science and Technology Innovation Fund (2023JJ12SN033)” to “Chao Wang”; and “Dalian Medical University Intensified Program of Wellness Interdisciplinary Research Cooperation Project Team Funding (JCHZ2023004)” to “Guixin Zhang” were erroneously removed. The corrected **Funding** statement is below:

