# Supplementary material for: Triterpenoids from Ganoderma lucidum inhibit cytochrome P450 enzymes interfering with the metabolic process of specific clinical drugs
Source: Front Pharmacol. 2024 Nov 15;15:1485209. doi: 10.3389/fphar.2024.1485209 (PMC11605156; doi:10.3389/fphar.2024.1485209)
Supplement: Supplementary file 1 [file DataSheet1.PDF]

Supplementary Material

Table S1. The information of probes for CYPs activity assay

CYP subtrype	Probe	Metabolite	Concentration
CYP1A2	Phenacetin	Deacetyl-phenacetin	40 μM
CYP2A6	Coumarin	7-hydroxy-coumarin	2 μM
CYP2C9	Diclofenac	4'-hydroxyl-diclofenac	4 μM
CYP2D6	Dextromethorphan	Demethyl-dextromethorphan	5 μM
CYP3A4	Midazolam	1'- hydroxyl-midazolam	5 μM
CYP2B6	Bupropion	4'-hydroxyl-bupropion	75 μM
CYP2C19	S-mephenytoin	4'-hydroxyl-S-mephenytoin	50 μM

Table S2. Information of clinical drugs

CYP subtype	Drugs	Metabolite	Concentration	Incubation time
CYP1A2	Melatonin	6-hydroxyl-melatonin	5 μM	30 min
	Clozaril	Demethyl-clozaril	10 μM	20 min
	Riluzole	Hydroxy-riluzole	5 μM	60 min
	Phenacetin	Deacetyl-phenacetin	40 μM	30 min
CYP2B6	Bupropion	4'-hydroxyl-bupropion	75 μM	30 min
	Artemisinin	Dihydroartemisinin	10 μM	30 min
	Sertraline	Demethyl-sertraline	5 μM	30 min
	Efavirenz	8-hydroxyl-efavirenz	20 μM	30 min
CYP2C9	Diclofenac	4'-hydroxyl-diclofenac	4 μM	30 min
	Valsartan	4-hydroxyl-valsartan	30 μM	30 min
	Glimepiride	Hydroxyl-glimepiride	50 μM	30 min
	Glibenclamide	Hydroxyl-glibenclamide	50 μM	30 min
CYP3A4	Midazolam	1'-hydroxyl-midazolam	5 μM	30 min
	Simvastatin	6'-β-hydroxyl-simvastatin	10 μM	30 min
	Diltiazem	Demethyl-diltiazem	10 μM	30 min
	Imatinib	Demethyl-imatinib	5 μM	30 min

Table S3. Mass spectrometry parameters for drugs metabolism investigation

Substrate	Metabolite	Ion pair	DP	EP	CE	CX
Phenacetin	Deacetyl-phenacetin	152.1→110.0	65	6	20	15
Coumarin	7-hydroxy-coumarin	163.0/119.0	12	22	15	15
Diclofenac	4'-hydroxyl-diclofenac	312.1→231.0	80	10	30	13
Dextromethorphan	Demethyl-dextromethorphan	258.1→157.0	60	9	35	15
Midazolam	1'-hydroxyl-midazolam	342.0-324.0	80	9	25	15
Bupropion	4'-hydroxyl-bupropion	256.0→238.0	60	10	20	16
S-mephenytoin	4'-hydroxyl-S-mephenytoin	235.0→150.0	10	25	16	15
Melatonin	6-hydroxyl-melatonin	249.2→190.2	50	10	20	13
Clozaril	Demethyl-clozaril	313.2→270.1	100	10	20	13
Riluzole	Hydroxy-riluzole	251.2→164.8	100	10	10	13
Artemisinin	Dihydroartemisinin	285.0→247.2	10	5	10	13
Sertraline	Demethyl-sertraline	292.3→159.2	50	2	50	13
Efavirenz	8-hydroxyl-efavirenz	330.0→258.0	-100	-2	-20	13
Simvastatin	6'-*β*-hydroxyl-simvastatin	437.5→303.3	40	10	10	13
Diltiazem	Demethyl-diltiazem	401.4→177.9	100	10	20	13
Imatinib	Demethyl-imatinib	479.6→394	10	10	50	13
Valsartan	4-hydroxyl-valsartan	452.3→235.1	50	2	20	13
Glimepiride	Hydroxyl-glimepiride	507.3→126.1	20	2	50	15
Glibenclamide	Hydroxyl-glibenclamide	511.0→367.1	20	5	25	15

Table S4. Mass spectrometry parameters for in vivo pharmacokinetic determination

Substrate	Ion pair	DP	EP	CE	CXP
Phenacetin	181.2→111.1	130	10	30	15
Bupropion	240.0→184.0	200	13	22	20
Diclofenac	295.0→250.0	80	-10	-15	-20
Midazolam	326.2→291.0	10	10	24	16

Table S5. The inhibitory effect of triterpenoids **1-66** against various CYPs.

No.	Residual activity (%)						
	**CYP1A2**	**CYP2D6**	**CYP3A4**	**CYP2A6**	**CYP2B6**	**CYP2C9**	**CYP2C19**
**1**	29.09±1.90	82.42±1.50	84.32±7.26	79.52±3.07	86.88±5.76	91.82±0.87	64.98±6.00
**2**	35.44±3.33	79.66±8.99	79.59±5.54	78.38±0.16	79.86±0.32	87.12±3.47	62.77±0.26
**3**	33.05±4.08	76.48±4.49	60.27±3.06	79.86±0.97	75.34±4.16	87.32±6.36	63.62±7.83
**4**	29.09±0.38	76.91±6.89	67.57±2.68	81.24±0.65	97.51±4.16	86.61±1.30	57.82±0.056
**5**	74.67±2.47	87.71±1.20	75.95±1.91	89.59±1.78	76.24±6.08	85.07±0.58	58.45±1.57
**6**	28.03±1.50	66.74±6.29	72.57±0.19	75.29±1.29	84.62±5.76	90.49±3.90	58.52±8.25
**7**	32.52±1.00	103.8±4.79	94.59±1.15	88.05±0.74	90.64±2.12	90.05±1.41	74.81±0.08
**8**	30.17±3.05	76.91±0.90	90.81±3.82	79.06±0.81	91.63±8.64	94.79±5.64	68.60±7.25
**9**	89.44±0.96	93.97±5.67	93.71±4.04	91.55±2.10	94.30±1.83	77.03±5.67	104.81±6.46
**10**	77.67±4.78	84.23±1.10	93.36±0.55	89.10±1.16	86.48±3.37	86.09±3.05	109.32±3.54
**11**	90.63±4.22	79.08±3.60	83.68±1.74	83.68±1.74	75.05±4.16	78.24±7.37	99.31±6.49
**12**	70.82±4.23	108.22±2.71	97.18±2.28	94.93±5.12	91.38±1.04	93.41±0.46	123.62±3.59
**13**	71.07±4.58	101.10±1.16	95.77±0.86	89.49±1.54	77.34±6.27	38.98±1.07	118.68±2.96
**14**	65.34±6.35	109.59±0.77	92.54±2.00	82.25±0.85	78.57±5.22	80.78±6.11	100.45±2.75
**15**	81.42±2.19	86.89±0.43	84.77±3.99	115.59±3.2	84.53±0.41	89.71±0.43	74.91±3.65
**16**	83.28±2.19	87.50±0.43	92.48±0.53	104.01±2.2	91.69±6.48	89.40±1.43	62.80±4.07
**17**	54.44±8.91	99.36±3.90	97.57±2.29	91.89±0.88	76.59±7.68	90.01±1.36	85.45±2.57
**18**	77.09±0.44	88.72±6.47	97.56±0.80	99.71±3.03	93.12±3.65	91.42±0.86	55.50±2.92
**19**	96.73±0.00	75.04±2.30	77.64±0.40	71.96±2.15	73.88±3.63	80.82±5.09	92.95±0.96
**20**	79.57±0.44	87.50±2.16	103.20±0.27	87.55±0.40	97.42±3.24	97.17±0.71	49.93±7.57
**21**	77.84±6.53	63.79±1.00	70.70±0.66	68.79±0.40	81.11±10.9	74.10±5.09	100.23±0.96
**22**	75.23±0.44	85.37±1.72	103.76±1.59	91.42±0.20	84.53±5.27	93.84±2.57	52.92±2.71
**23**	78.98±8.77	85.77±4.68	85.47±6.21	99.75±2.28	73.99±0.62	68.85±4.39	75.41±1.44
**24**	105.49±2.96	76.47±5.39	79.31±3.01	79.31±3.01	57.95±5.14	50.57±3.67	128.47±15.0
**25**	103.75±18.9	68.22±0.44	30.07±1.54	70.08±2.15	56.78±2.04	31.96±2.74	39.21±4.83
**26**	93.97±3.49	85.82±3.22	68.01±1.43	93.52±1.46	91.06±2.30	73.11±1.01	107.03±3.59
**27**	58.60±10.23	98.90±4.26	77.02±5.13	93.00±2.05	68.23±7.31	75.38±3.05	97.09±8.56
**28**	92.05±5.42	73.42±5.01	85.86±1.43	92.64±1.46	90.65±6.32	91.72±3.04	127.28±3.49
**29**	101.10±10.4	88.61±0.72	87.37±3.57	100.15±4.1	96.75±10.3	94.58±2.75	106.65±5.39
**30**	97.52±1.31	92.99±2.16	89.10±2.13	93.71±0.61	91.12±0.81	95.86±3.42	74.72±3.91
**31**	53.74±1.44	102.75±3.30	103.92±1.72	86.17±0.44	71.16±4.24	80.99±0.51	94.81±4.46
**32**	77.40±1.75	94.21±3.88	81.77±3.99	96.42±2.02	84.81±5.67	87.79±0.86	68.60±3.91
**33**	87.40±5.04	88.10±2.86	78.11±0.95	94.26±0.83	104.47±2.8	94.68±2.89	106.35±3.07
**34**	110.14±2.32	96.46±1.79	70.20±1.67	94.11±2.71	77.48±4.25	51.43±4.77	81.54±3.49
**35**	86.30±1.16	90.89±6.09	57.07±0.24	100.15±2.5	83.33±2.87	44.79±0.29	67.26±0.85
**36**	81.92±5.81	72.66±10.38	55.39±1.19	87.04±1.46	88.62±6.90	82.92±2.75	84.38±0.53
**37**	87.95±3.49	103.54±3.22	60.10±3.57	104.12±1.4	94.31±3.45	94.79±3.33	97.61±4.86

**38**	120.37±10.4	108.42±14.3	118.07±13.9	63.04±3.71	111.77±7.3	100.77±0.7	99.58±7.03
**39**	79.71±6.66	94.25±3.63	81.64±3.87	91.77±11.0	89.40±8.43	87.35±2.32	96.14±2.25
**40**	64.45±3.90	98.06±1.54	87.89±7.18	88.89±6.98	75.83±7.96	92.04±8.28	101.82±9.64
**41**	88.77±0.00	99.75±0.00	54.04±1.67	93.81±0.62	93.90±0.57	87.83±1.30	71.00±7.19
**42**	43.77±4.18	103.18±3.30	96.76±4.20	83.68±8.67	89.14±3.71	62.82±2.72	79.30±0.38
**43**	62.44±5.08	84.24±14.04	113.22±16.2	60.67±4.10	99.02±9.29	99.68±2.81	87.05±10.57
**44**	86.53±1.20	99.00±3.81	91.24±1.70	81.12±5.93	85.55±4.31	97.35±2.50	83.70±20.65
**45**	48.24±4.71	93.69±8.49	109.74±3.23	90.11±2.87	82.99±3.05	73.55±6.44	70.26±4.05
**46**	97.86±4.74	94.14±0.61	94.40±2.82	89.06±3.22	92.82±5.38	100.20±1.4	113.46±9.56
**47**	73.05±5.97	108.62±5.49	95.70±8.29	92.18±4.66	99.01±9.83	106.09±0.9	110.91±3.86
**48**	95.14±8.37	96.48±6.02	97.63±2.24	93.00±2.22	93.20±3.24	90.14±6.22	112.22±3.57
**49**	61.36±0.46	93.24±5.93	93.36±2.76	92.18±1.16	86.75±3.75	68.85±3.31	105.00±4.50
**50**	62.18±2.53	93.94±6.92	98.83±1.66	89.71±3.49	77.48±4.68	96.49±3.97	116.59±7.39
**51**	58.01±2.08	89.51±3.30	94.14±7.18	86.01±4.07	77.81±7.96	99.77±6.62	109.77±3.54
**52**	88.47±3.59	94.97±5.72	96.63±1.70	91.45±1.90	86.88±1.44	85.69±5.91	99.14±9.67
**53**	78.33±2.19	92.68±6.04	87.03±0.27	100.57±1.0	99.14±4.86	87.69±0.71	68.63±15.66
**54**	47.09±3.09	108.69±3.90	100.00±8.41	82.64±2.79	84.08±1.32	88.45±0.51	88.34±3.63
**55**	71.10±3.67	86.79±5.16	71.41±3.09	74.81±0.70	77.81±6.09	96.25±6.96	104.32±8.04
**56**	56.01±0.69	86.87±1.32	82.81±1.10	72.30±1.11	84.11±0.94	92.51±0.99	109.09±6.43
**57**	67.86±1.38	86.25±7.69	81.25±2.21	79.38±2.50	76.16±2.81	94.38±4.97	90.91±0.64
**58**	63.31±1.38	87.33±0.66	77.19±0.99	73.00±0.12	80.13±1.87	83.14±0.33	97.73±1.93
**59**	78.02±3.50	82.32±7.76	78.38±4.52	90.56±2.63	75.64±3.24	72.35±0.43	67.90±3.03
**60**	122.37±13.1	99.06±1.27	106.17±8.24	59.78±4.43	102.02±7.6	102.02±7.6	68.76±4.05
**61**	62.66±0.46	79.07±5.20	103.20±10.1	100.45±8.2	78.37±1.82	84.65±5.77	95.68±7.39
**62**	71.98±2.26	72.07±2.30	61.84±8.45	63.72±0.87	41.54±7.21	69.06±9.50	100.00±0.00
**63**	84.38±0.77	98.73±0.00	106.90±0.24	102.65±1.4	91.46±12.0	95.30±0.29	71.66±0.73
**64**	135.21±7.02	78.29±1.77	77.93±0.00	77.93±0.00	67.10±1.66	59.73±4.70	126.87±8.09
**65**	90.03±1.16	91.58±1.30	95.20±0.00	92.86±3.79	91.54±5.91	92.33±1.02	97.95±0.32
**66**	60.45±2.57	87.60±0.79	90.54±8.87	58.85±4.63	80.75±6.48	80.75±6.48	57.73±5.64

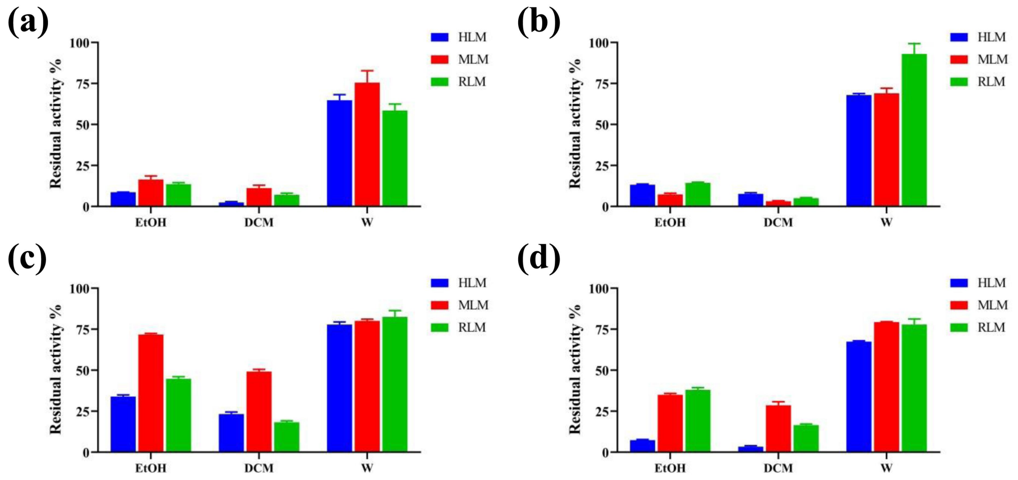

Figure S1. The inhibitory effects of different extracts of *Ganoderma lucidum* against CYPs from various species. (a) CYP 2C9, (b) CYP 3A4, (c) CYP 1A2, (d) CYP 2B6. HLM (human liver microsome), MLM (mouse liver microsome), RLM (rat liver microsome). EtOH (Ethanol extract), DCM (Dichloromethane extract), W (Water extract).

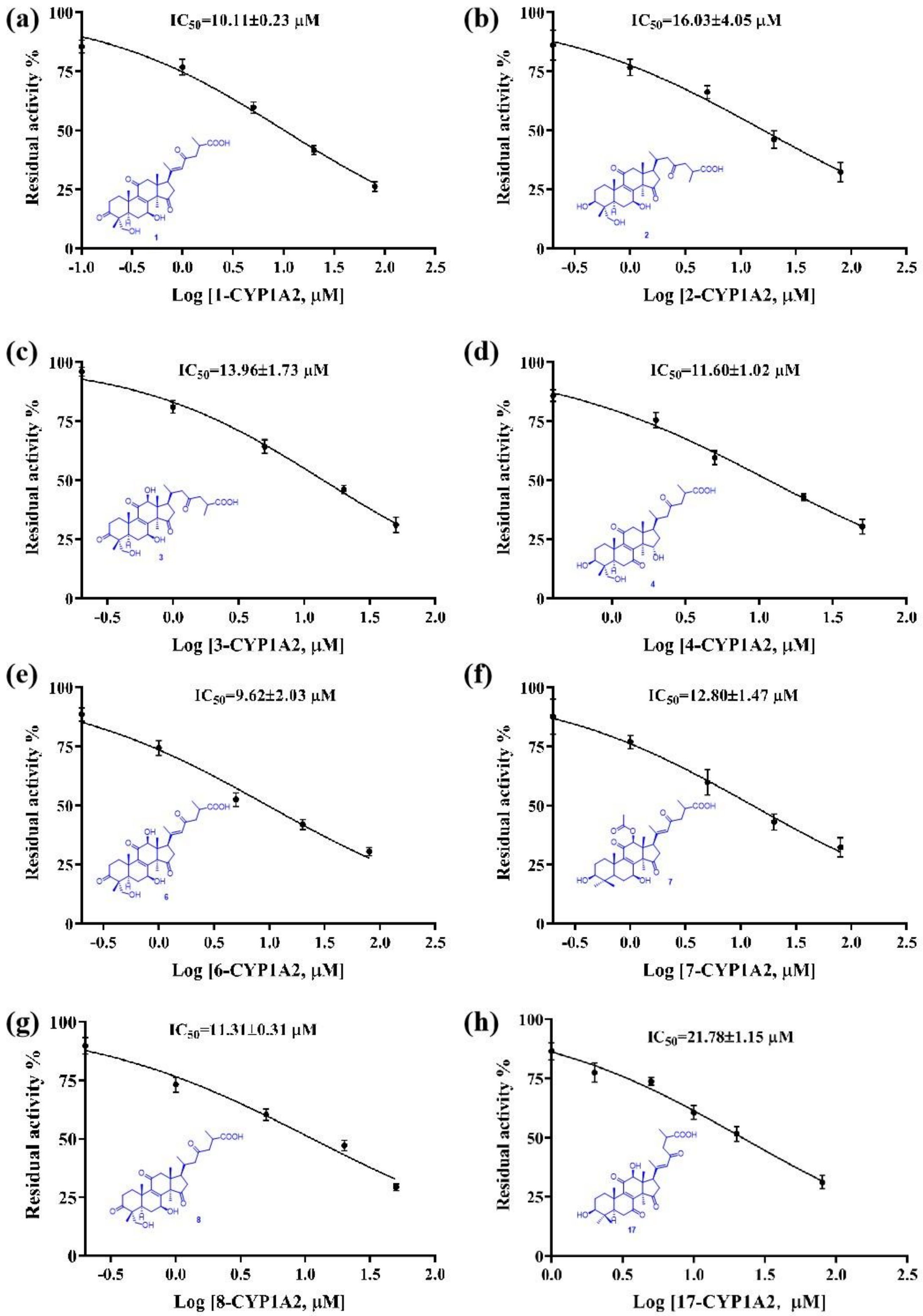

Figure S2. The IC_{50} determination of triterpenoids **1**, **2**, **3**, **4**, **6**, **7**, **8**, and **17** against CYP 1A2.

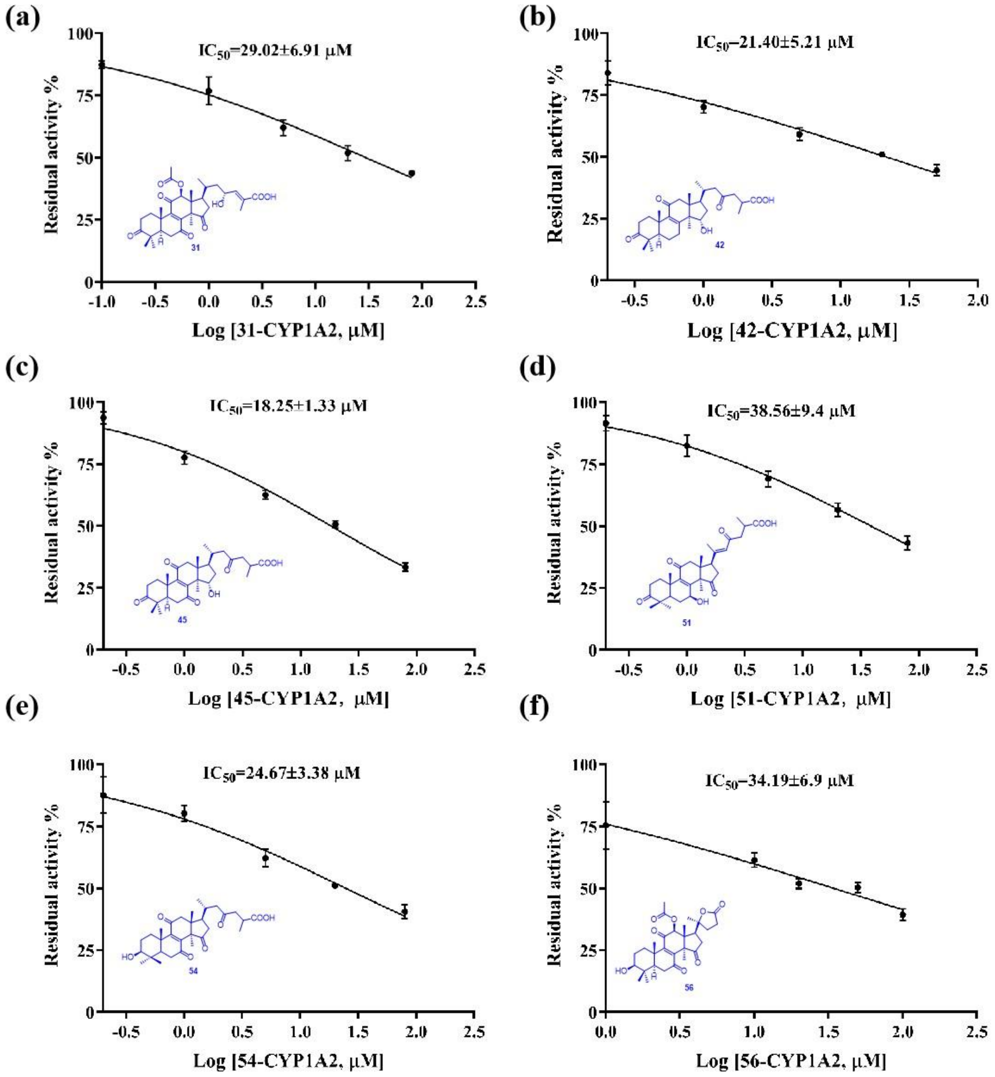

Figure S3. The IC_{50} determination of triterpenoids **31**, **42**, **45**, **51**, **54**, and **56** against CYP 1A2.

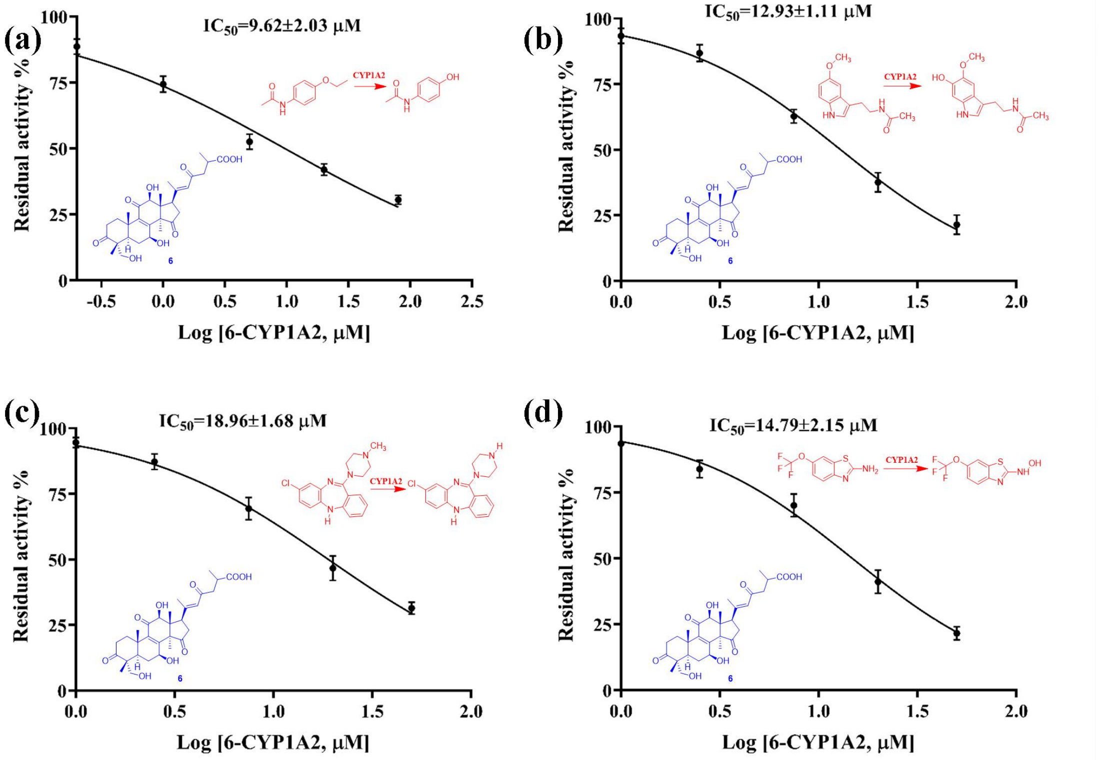

Figure S4. The IC_{50} determination of triterpenoid **6** inhibiting the metabolism of clinical drugs mediated CYP 1A2. (a) Phenacetin, (b) Clozapine, (c) Melatonin, (d) Riluzole.

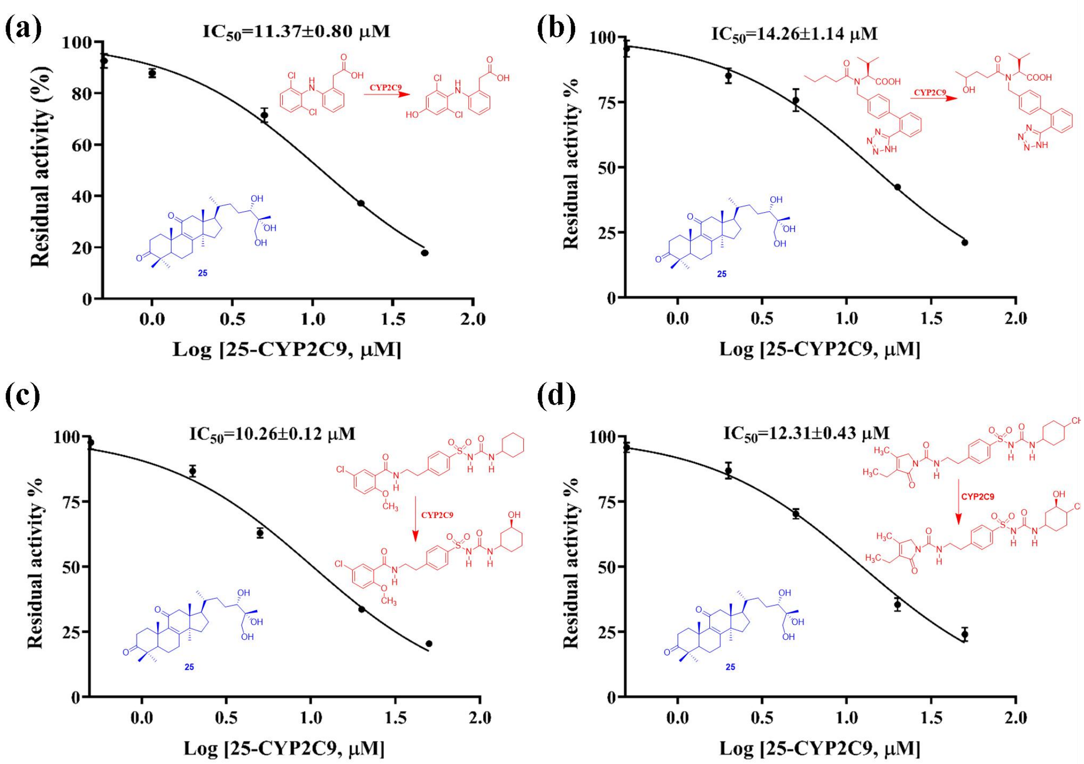

Figure S5. The IC_{50} determination of triterpenoid **25** inhibiting the metabolism of clinical drugs mediated CYP 2C9. (a) Diclofenac, (b) Valsartan, (c) Glibenclamide, (d) Glimepiride.

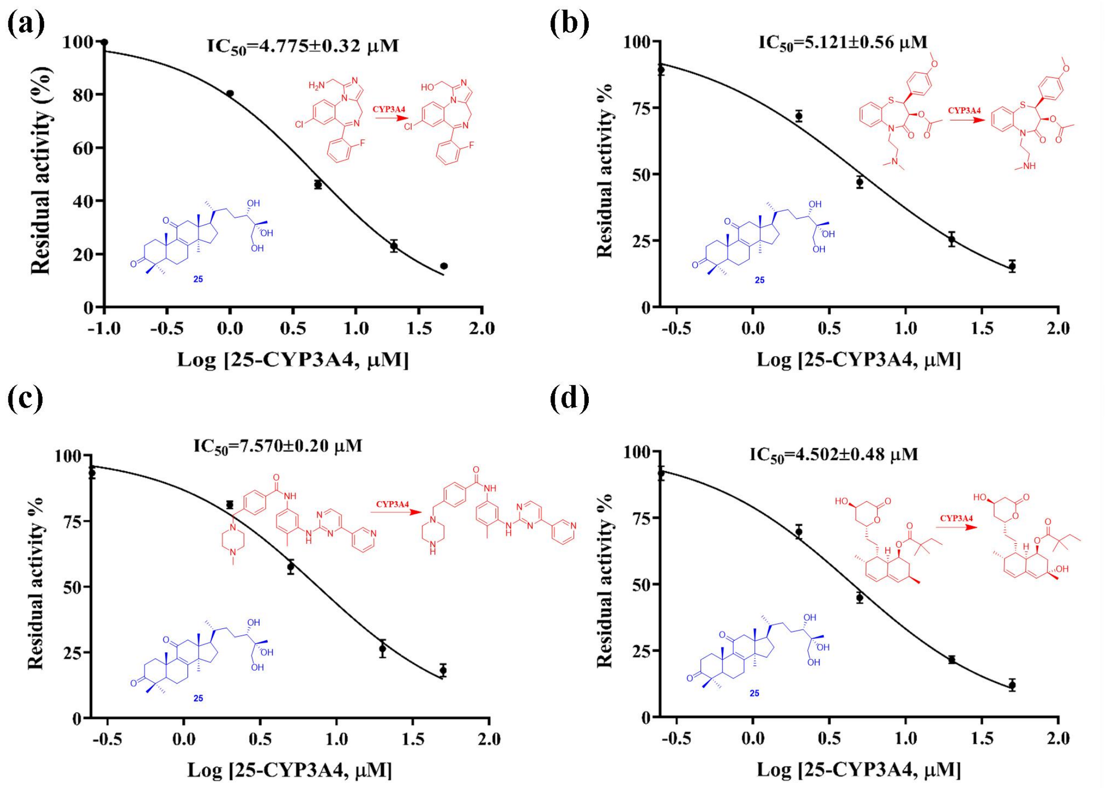

Figure S6. The IC_{50} determination of triterpenoid **25** inhibiting the metabolism of clinical drugs mediated CYP 3A4. (a) Midazolam, (b) Diltiazem, (c) Imatinib, (d) Simvastatin.

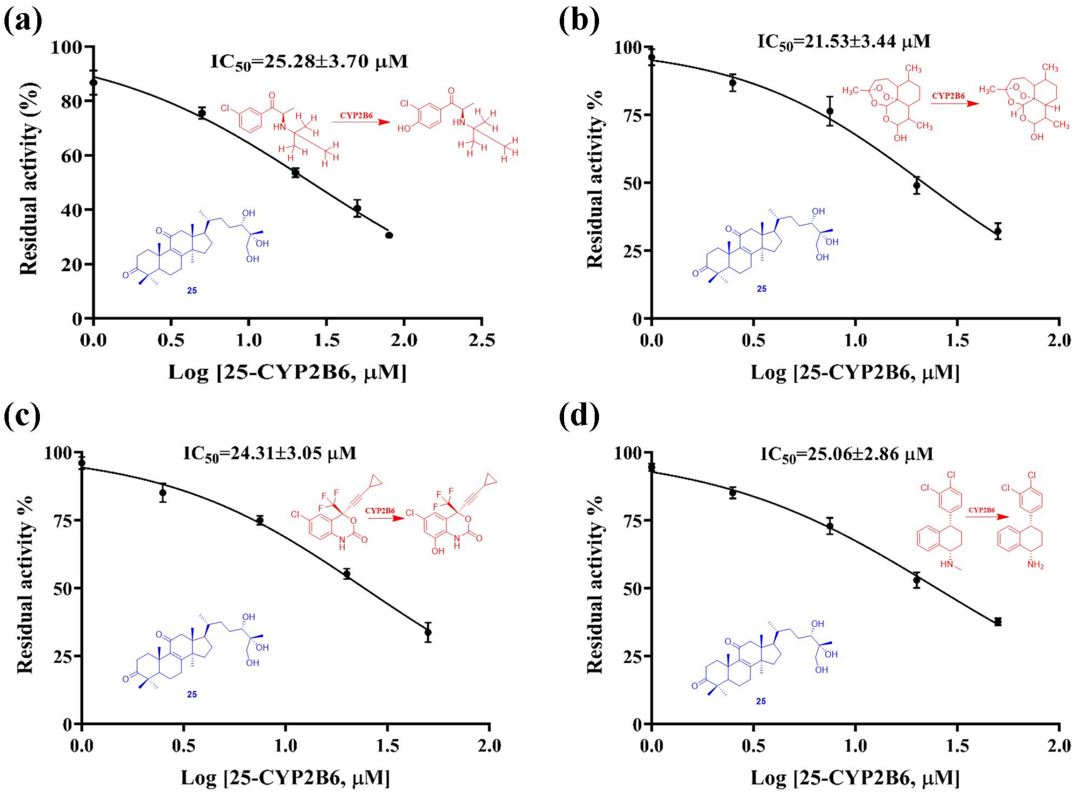

Figure S7. The IC_{50} determination of triterpenoid **25** inhibiting the metabolism of clinical drugs mediated CYP 2B6. (a) Bupropion, (b) Artemisinin, (c) Efavirenz, (d) Sertraline.
